# Carnitine Requires Choline to Exert Physiological Effects in *Saccharomyces cerevisiae*

**DOI:** 10.3389/fmicb.2018.01362

**Published:** 2018-07-02

**Authors:** Michelle du Plessis, Jaco Franken, Florian F. Bauer

**Affiliations:** Department of Viticulture and Oenology, Institute for Wine Biotechnology, Stellenbosch University, Stellenbosch, South Africa

**Keywords:** carnitine, choline, oxidative stress, *CHO2*, *OPI3*, *Saccharomyces cerevisiae*

## Abstract

L-Carnitine is a key metabolite in the energy metabolism of eukaryotic cells, functioning as a shuttling molecule for activated acyl-residues between cellular compartments. In higher eukaryotes this function is essential, and defects in carnitine metabolism has severe effects on fatty acid and carbon metabolism. Carnitine supplementation has been associated with an array of mostly beneficial impacts in higher eukaryotic cells, including stress protection and regulation of redox metabolism in diseased cells. Some of these phenotypes have no obvious link to the carnitine shuttle, and suggest that carnitine has as yet unknown shuttle-independent functions. The existence of shuttle-independent functions has also been suggested in *Saccharomyces cerevisiae*, including a beneficial effect during hydrogen peroxide stress and a detrimental impact when carnitine is co-supplemented with the reducing agent dithiothreitol (DTT). Here we used these two distinct yeast phenotypes to screen for potential genetic factors that suppress the shuttle independent physiological effects of carnitine. Two deletion strains, Δ*cho2* and Δ*opi3*, coding for enzymes that catalyze the sequential conversion of phosphatidylethanolamine to phosphatidylcholine were identified for suppressing the phenotypic effects of carnitine. Additional characterisation indicated that the suppression cannot be explained by differences in phospholipid homeostasis. The phenotypes could be reinstated by addition of extracellular choline, but show that the requirement for choline is not based on some overlapping function or the structural similarities of the two molecules. This is the first study to suggest a molecular link between a specific metabolite and carnitine-dependent, but shuttle-independent phenotypes in eukaryotes.

## Introduction

In eukaryotes, L-carnitine has been extensively studied for its role in the carnitine shuttle. The shuttle is required for the transport of activated acyl residues between cellular organelles, and in particular between the mitochondria, the peroxisomes and the cytosol (Vaz and Wanders, 2002). More recent studies have investigated the effect of carnitine supplementation during cellular stress (Silva-adaya et al., 2008; Li et al., 2012; Sachan et al., 2012). Supplementation of carnitine generally appears to have beneficial and potentially therapeutic effects for a range of diseases, including neurodegenerative and metabolic disorders (Calabrese et al., 2006). In some instances these effects can be directly linked to the shuttling activity, whereas in other cases, the mechanism by which these effects are achieved remain unclear. Carnitine has been shown to alleviate the damaging effect of reactive oxygen species (Calabrese et al., 2006). These cellular effects are largely conserved in eukaryotes, including the eukaryotic model organism, *Saccharomyces cerevisiae* (Franken et al., 2008; Franken and Bauer, 2010). Previous studies in our laboratory have specifically illustrated that carnitine supplementation results in improved growth for cultures stressed with hydrogen peroxide (Franken and Bauer, 2010). Interestingly, it was also found that carnitine increased the toxic effects of the thiol reducing agent DTT. Both of these outcomes were found to occur independently of the carnitine shuttle (Franken et al., 2008). Although several interactions of carnitine with cytoprotective genes, membranes and membrane associated proteins have been suggested (Arduini et al., 1993; Butterfield and Rangachari, 1993; Calabrese et al., 2006), no biological function that would be unrelated to the carnitine shuttle has thus far been implicated on a molecular level.

To search for potential molecular targets of shuttle independent activities of carnitine, this study used a genetic approach by screening for mutations that would suppress shuttle-independent phenotypes.. Three deletion mutants were identified, the general stress related transcription factor Yap1p and two enzymes required for the synthesis of phosphatidylcholine (PC), *Cho2*p and *Opi3*p. Yap1p has previously been identified as essential for the impact of carnitine during oxidative stresses (Franken et al., 2008; Franken and Bauer, 2010). The focus of this work was therefore to specifically characterize the role of *OPI3* and *CHO2*.

**FIGURE 1:**
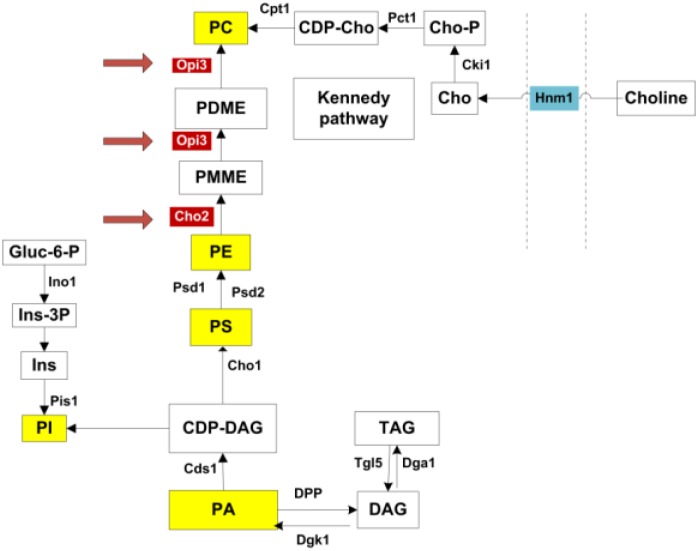
Phospholipid synthesis pathways. All major phospholipids are highlighted in yellow. *Opi3*p and *Cho2*p, highlighted in red, catalyze methylation reactions in the synthesis of PC from PE. PC may also be synthesized via the Kennedy pathway when external choline is taken up through the transporter Hnm1p. PA, phosphatidic acid; DAG, diacylglycerols; TAG, triacylglycerols; CDP-DAG, CDP-diacylglycerol; PI, phosphatidylinositol; Ins, inositol; Ins-3P, inositol 3-phosphate; Gluc-6-P, glucose-6-phosphate; PS, phosphatidylserine; PE, phosphatidylethanolamine; PMME, phosphatidylmonomethylethanolamine; PDME, phosphatidyldimethylethanolamine; PC, phosphatidylcholine; CDP-cho, CDP-choline; Cho-P, choline phosphate; Cho, choline. Adapted from (Henry et al., 2012).

Intriguingly the two enzymes *Cho2*p and *Opi3*p, are required to catalyze the final, sequential steps in the synthesis of phosphatidylcholine (PC) from phosphatidylethanolamine (PE) (Henry et al., 2012). As illustrated in **Figure 1**, this represent one metabolic route toward PC synthesis, referred to as the CDP-DAG pathway. An alternative route, the Kennedy pathway, requires the uptake of external choline to serve as precursor in the synthesis of PC (Henry et al., 2012). PC is a major membrane phospholipid which is thought to account for approximately 50% of the phospholipids within the cell and is important in membrane structure (Boumann et al., 2003, 2006). With recent advances in technology enabling more precise analysis of lipids, the study of lipid composition, homeostasis and regulation has become a central focus in understanding the cells adaption to various metabolic and environmental conditions. In particular, various studies have investigated the regulation of lipid composition under stress conditions and how this influences cell survival (Beales, 2004; Balogh et al., 2013; Henderson et al., 2013).

The genetic link between the carnitine-dependent phenotypes and suppressor mutations in genes involved in PC synthesis suggests that carnitine may either impact or require specific membrane lipid composition or impact on membrane homeostasis. For this purpose, the lipidome of cells was investigated in the mutants and in response to treatment with carnitine, choline, hydrogen peroxide and DTT. The data, however, suggest no impact of carnitine on lipid composition. The data also show that the carnitine-dependent phenotypes were restored by providing extracellular choline. These evidence suggests that either phosphatidylcholine or its precursor, choline is required for carnitine to have an impact on cellular resistance to oxidative stresses, and provides the first genetic and metabolic link between carnitine-dependent, but shuttle independent phenotypes and other metabolic pathways in eukaryotes.

## Materials and Methods

### Strains and Media

The strains used in this study were obtained from Euro scarf (Frankfurt, Germany) and are listed in **Table 1** (Giaever and Nislow, 2014^1^). Strains were grown in minimal SCD media (2% glucose, 0.67% YNB without amino acids (Difco). Amino acids were supplemented as required by the different strains at a concentration previously described (Ausubel et al., 1994). The DTT, carnitine and choline used in this study were obtained from Sigma. Strains used for the mutant screens were of the S288c genetic background and obtained from Euroscarf, with all gene disruptions done by replacement of the designated open reading frame (ORF) by the Geneticin resistance cassette (ORF::KanMX4).

**Table 1:** Strains used in this study.

Yeast strains	Genotype	Ref
BY4742	MATα *his3 lys2 leu2 ura3*	Euroscarf
BY4741	MATa *his3 lys2 leu2 ura3*	Euroscarf
BY4742Δ*yap1*	MATα *his3 lys2 leu2 ura3*Δ*yap1*::kanMX	Euroscarf
BY4742Δ*opi3*	MATα *his3 lys2 leu2 ura3*Δ*opi3*::kanMX	Euroscarf
BY4742Δ*cho2*	MATα *his3 lys2 leu2 ura3*Δ*cho2*::kanMX	Euroscarf

### Spotting Assay

Media was made up on the day prior to analysis and 8 mM DTT or 1 mM hydrogen peroxide with or without 5 mM carnitine was added where appropriate as previously described (Franken and Bauer, 2010). Strains were grown overnight at 30°C in 5 ml SCD. Cultures were then diluted to OD_600nm_ of 1 and five sequential ten times dilutions were made in a microtiter plate. These were then spotted onto plates in 10 μl volumes. Plates were incubated at 30°C.

### Liquid Growth Assays

Strains were grown in 5 ml SCD overnight at 30°C and then inoculated into a second round of starter cultures in 10 ml volumes and grown overnight. The cultures were then spun down and resuspended in 10 ml medium with and without carnitine and/or choline at a final OD_600nm_ of 0.1. Cultures were grown until mid-log phase and then treated with hydrogen peroxide or DTT. The optical density of the cultures was monitored over a period of 30 h. For survival assays, cultures were plated out just before and for several time points after addition of hydrogen peroxide.

### Lipid Extraction

Prior to lipid extraction cultures were grown to an OD_600nm_ of 0.8 unless otherwise specified and then treated with DTT or hydrogen peroxide. The cultures were then spun down at 6000 RPM for 1 min, washed with distilled water, weighed and frozen at -80°C. Lipid extraction was carried out as previously described (Ejsing et al., 2009) with some alterations. Cultures were thawed prior to extraction, resuspended in 200 μL NH_4_HCO_3_ (150 mM, pH 8) and 300 μL glass beads were added. Cells were disrupted by vortexing for 3 min. For the first extraction, 1 ml chloroform: methanol (17:1) was added and samples were placed on a shaker for 2 h at 30°C. The organic phase was then removed and placed in a new tube and the aqueous phase was re-extracted with 1 ml chloroform: methanol (2:1) and 0.5% formic acid which was again carried out on a shaker at 30°C for 2 h. Thereafter, the organic phase was collected and added to the first. Samples were then dried, resuspended in chloroform: methanol (1:2) and stored at -20 until analysis. The chloroform, methanol and formic acid used were obtained from Sigma.

### HILIC-ESI-MS

The analysis of the lipid composition of cultures was adapted from a method developed by Castro-Perez et al. (2010). Analysis was performed on a Waters Acquity UPLC (Waters, Milford, MA, United States), connected to a Waters Synapt G2 quadrupole time-of-flight (QTOF) mass spectrometer fitted with an electrospray ionization probe. Compounds were separated on a Waters HSS T3 column, 2.1×150 mm, with a 1.8 μm particle size at a flow rate of 0.32 ml/min. The injection volume was 5 μl. Lipids were analyzed both in positive (PC, PE, and TAG) and negative (PI, PS, and PA) mode using a capillary voltage of 2.5 kV, cone voltage of 15 V, desolvation gas (nitrogen) flow rate of 650 L/hr and temperature at 275°C. Solvent A was made up of 0.6 g/L ammonium formate in 60% acetonitrile and solvent B consisted of isopropanol-acetonitrile (9:1) with 0.6 g/L ammonium formate. For the first 11 min of the run 60% solvent A was used. Thereafter, this was changed to 100% solvent B. At 14 min it was returned to 60% A. The total run time was 17 min. Data was acquired in MS^E^ mode which consists of a lower collision energy scan (*m/z* 150–1500 at 6 V) and a higher energy scan (*m/z* 40 to 1500, collision energy ramp from 20 to 60 V) every 0.2 s. The instrument was calibrated with sodium formate and leucine enkephalin was used as lock spray for accurate mass determinations. The resulting data was analyzed using Masslynx version 4.1.

### Genome-Wide Screen for Mutants With Reduced Responsiveness to Carnitine Supplementation

A genome-wide deletion strain screen for loss of responsiveness to carnitine supplementation during stress conditions was carried out using the haploid BY4741 deletion collection (*ORF*::kanMX4). Strains were pinned in 384 format on media containing 2 mM DTT; 2 mM DTT + 1 g/L carnitine; 0.4 mM H_2_O_2_; 0.4 mM H_2_O_2_ + 1 g/L carnitine and SCD as control using a benchtop RoToR HDA robot (Singer Instruments, United Kingdom) with default settings. After 2 and 4 days, respectively, colonies were scanned using a desktop scanner. Images were evaluated by comparing of colony size visually between carnitine supplemented and unsupplemented plates. Mutants that display slow growth phenotype were excluded from the analysis. A total of 4,311 gene disruption mutants were spotted and analyzed. Rescreening to confirm the phenotypes of selected mutants was performed by spotting corresponding disruption mutants from the BY4742 genetic background on standard agar plates as described in the spotting assay above.

## Results

### Screening for Single Disruption Mutants That Are Less Responsive to the Effect of Carnitine Supplementation During Oxidative Stress

**FIGURE 2:**
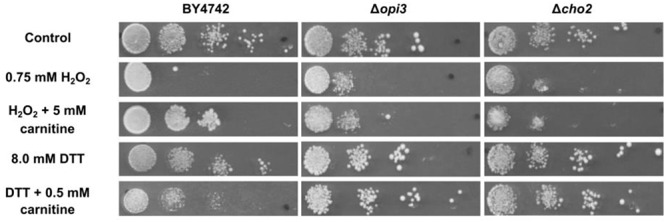
Plate assays for response of wild type BY4742 and the mutants; Δ*cho2* and Δ*opi3* when stressed with DTT or hydrogen peroxide in the presence and absence of carnitine.

The phenotype screening yielded nine mutants that were selected for being less responsive to carnitine in the presence of DTT. A further 49 mutants were selected for decreased responsiveness to carnitine on plates that contain H_2_O_2_as stress inducing agent. These two sets of mutants were re-streaked and spotted again on both DTT and peroxide containing plates with and without carnitine supplementation. From these mutants only three displayed consistent and repeatable suppression of both carnitine related phenotypes. In other words, these mutants did not display increased resistance to H_2_O_2_ and did not display increased sensitivity in the presence of DTT. The three mutants that were identified are deletion strains of the transcription factor *YAP1*, and two strains deleted for genes that encode enzymes involved in the synthesis of phosphatidylcholine, namely *CHO2* and *OPI3*. All three of the identified genes resulted in an almost complete loss of responsiveness to carnitine (**Figure 2**. for Δ*cho2* and Δ*opi3*; Franken et al., 2008; Franken and Bauer, 2010 for Δ*yap1*).

The mitochondrial Cyc3p that was previously identified for its requirement in the carnitine phenotypes (Franken and Bauer, 2010) was not isolated in this screen since it was excluded as part of the slow growing strains as mentioned in the methods section.

The transcription factor Yap1p was previously shown to block both the DTT and H_2_O_2_ carnitine associated phenotypes (Franken and Bauer, 2010). This transcription factor has been extensively characterized for its role as a transcriptional regulator of the general oxidative stress response. Two additional transcriptional factors, Msn2p and Msn4p, are also considered to be central transcriptional regulators of the yeast’s oxidative stress response (De la Torre-Ruiz et al., 2010). A BY4742Δ*msn2*Δ*msn4* was used to test if the blockage of the cell’s oxidative stress response via loss of Msn2p and Msn4p would result in a similar loss of response to carnitine supplementation. The data indicated that the effect of carnitine supplementation is exactly the same in the Δ*msn2*Δ*msn4* double mutant as the effect observed in wild type cultures (data not shown) on both DTT and H_2_O_2_ containing plates. This result indicated that the carnitine effect is not dependent on the cells general ability to activate the oxidative stress response, but that the phenotype is specifically dependent on the presence of Yap1p. The carnitine phenotypes therefore either involves a specific function of the Yap1p protein or a specific subset of transcriptional targets that is specific to Yap1p and not to Msn2p and Msn4p. The deeper characterisation of the role of Yap1p, however, falls outside of the scope of the current publication. The interest of this manuscript is to specifically provide a more in depth characterisation of the phenotypes associated with *Cho2*p and *Opi3*p.

### Characterisation of the Carnitine Related Phenotypes of Δ*cho2* and Δ*opi3*

To confirm and characterize the carnitine phenotypes of the wild type and mutant strains, cultures were grown to O.D ∼ 1.0 in liquid minimal media (SCD) and spotted on solid media (SCD) containing hydrogen peroxide or DTT in the presence or absence of carnitine. The assays show reduced growth for all strains on plates containing 0.75 mM hydrogen peroxide (**Figure 2**). In the presence of 5 mM carnitine, a protective effect is clearly apparent for the wild type, but not in the two lipid mutants. The addition of 8 mM DTT to the media also resulted in decreased growth in all strains. In confirmation of previous findings, the growth of the WT is further reduced upon co-supplementation of carnitine and DTT (Franken and Bauer, 2010). In the case of Δ*cho2* or Δ*opi3*, however, the increased toxicity of the DTT carnitine combination is almost entirely absent (**Figure 2**).

The inability of the mutants which are involved in the synthesis of phosphatidylcholine to respond to carnitine treatment suggests a link between the carnitine-dependent phenotypes and phospholipid metabolism. Furthermore, the identification of two enzymes catalyzing successive reactions in the same metabolic branch provides a strong indication that the observed carnitine phenotypes are in some manner dependent on the metabolism or function of either choline or phosphatidylcholine. It is of additional interest that carnitine and choline share a striking similarity on a structural chemical level, both containing a trimethyl-ammonium moiety, and both compounds also make use of at least one shared uptake mechanism through the transporter Hmn1p (Aouida et al., 2013). In this context, it is possible that the absence of choline in the mutants impacted the transport of carnitine, explaining the suppression phenotypes of the *OPI3* and *CHO2* deletions. For this reason, the deletion mutants of the *HMN1* gene, encoding the carnitine transporter, and of a gene previously identified as a carnitine transporter, but now rather thought to act as a sensor and regulator of transport, *AGP2* (Aouida et al., 2013), were assessed. No suppression of the carnitine-dependent phenotypes was observed in these mutants (data not shown).

**FIGURE 3:**
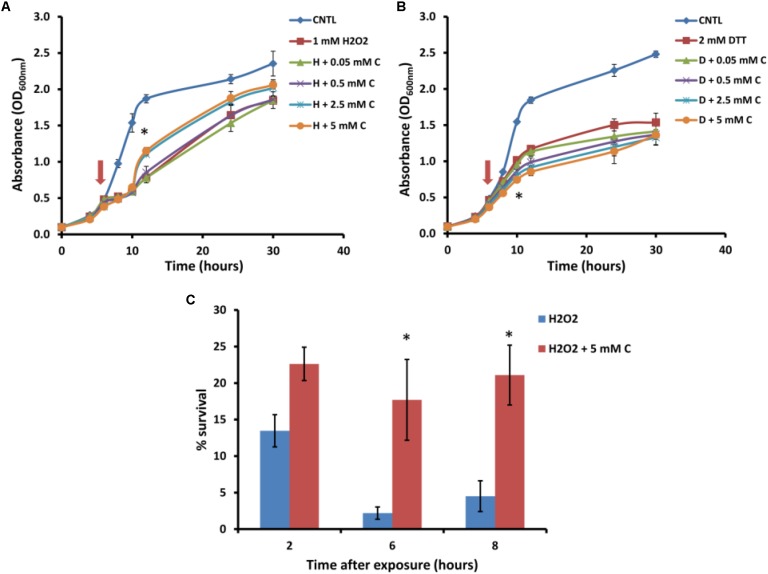
Response of BY4742 strain to oxidative stress induced by hydrogen peroxide and DTT in the presence and absence of carnitine (C). Liquid analysis monitoring growth before and after exposure to hydrogen peroxide **(A)**. Liquid analysis monitoring growth before and after exposure to DTT **(B)**. The time at which either DTT or hydrogen peroxide was added is indicated by an arrow. Percentage survival of cultures grown in the presence or absence of carnitine after exposure to hydrogen peroxide **(C)**. All experiments were done in triplicate with error bars representing standard deviation (^∗^*p* < 0.05; 5 mM carnitine vs. H2O2 or DTT).

For further analysis of the suppression of carnitine-dependent phenotypes by the two lipid mutants, the stress phenotypes observed on plates were also evaluated in liquid medium in order to provide better quantification of the impact and to obtain a clearer distinction between the phenotypes (**Figure 3**). The concentrations of carnitine, DTT and hydrogen peroxide, as well as the timing of treatment with the redox stress inducing chemicals were re-optimized for such liquid media conditions. Addition of hydrogen peroxide at a concentration of 1 mM to the wild type cultures inhibited growth for a period of time after which cultures resumed growth. Cultures supplemented with carnitine recovered faster than those without carnitine (**Figure 3A**). This effect was dependent on the concentration of carnitine, with 50 and 500 μM providing no positive effects and the maximum response reached at 2.5 mM carnitine. In addition, plate counts of samples that were plated on YPD solid media (2, 6, and 8 h after exposure to H_2_O_2)_ show that cultures containing carnitine had a higher survival percentage than those without carnitine (**Figure 3C**).

The treatment of cultures with DTT at 2 mM resulted in cessation of growth from which the cells did not recover within the time period monitored (**Figure 3B**). Cultures which contained carnitine in addition to DTT, however, plateaued at a lower optical density than those with only DTT. The effect for carnitine, as with hydrogen peroxide, occurred in a concentration dependent manner. However, an effect for carnitine in the DTT exposed cultures were visible at a lower concentration (500 μM) than with hydrogen peroxide.

**FIGURE 4:**
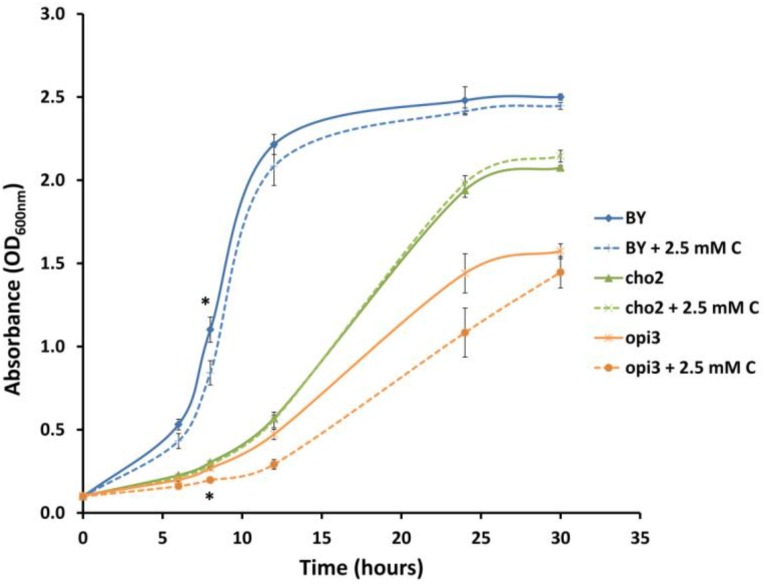
Carnitine supplementation (C) leads to slightly reduced growth of wild type (BY) and Δ*opi3* cultures but not for Δ*cho2*. All experiments were done in triplicate with error bars representing standard deviation (^∗^*p* < 0.05 vs. untreated culture).

In order to investigate whether carnitine supplementation had any effect on the growth of cultures, and also to determine whether it is capable of compensating for the dependence of the mutants on supplemented choline, the wild type and mutant cultures were inoculated into media with and without 2.5 mM carnitine (**Figure 4**). It was found that wild type cultures grew slightly slower in the presence of carnitine in the early stages of growth but caught up with the un-supplemented cultures toward the end of the log phase. However, carnitine did not compensate for the absence of choline in the mutant cultures. The growth impeding effect of carnitine was even stronger in Δ*opi3* than the wild type. However, carnitine supplementation had no measurable effect on the growth of Δ*cho2.*

### Effect of Choline Addition on Carnitine Phenotypes

In addition to the synthesis of PC via the CDP-DAG pathway, this phospholipid can also be produced through the Kennedy pathway when cells are supplemented with choline (Dowd et al., 2001; **Figure 1**). It was therefore of interest to establish whether choline supplementation to Δ*cho2* and Δ*opi3* mutant cells, in restoring PC synthesis, would restore the effects of carnitine. In addition, when taking the similarities between carnitine and choline into account, it was also important to establish if choline supplementation to wild type cells would simulate similar phenotypes to those linked with carnitine supplementation.

**FIGURE 5:**
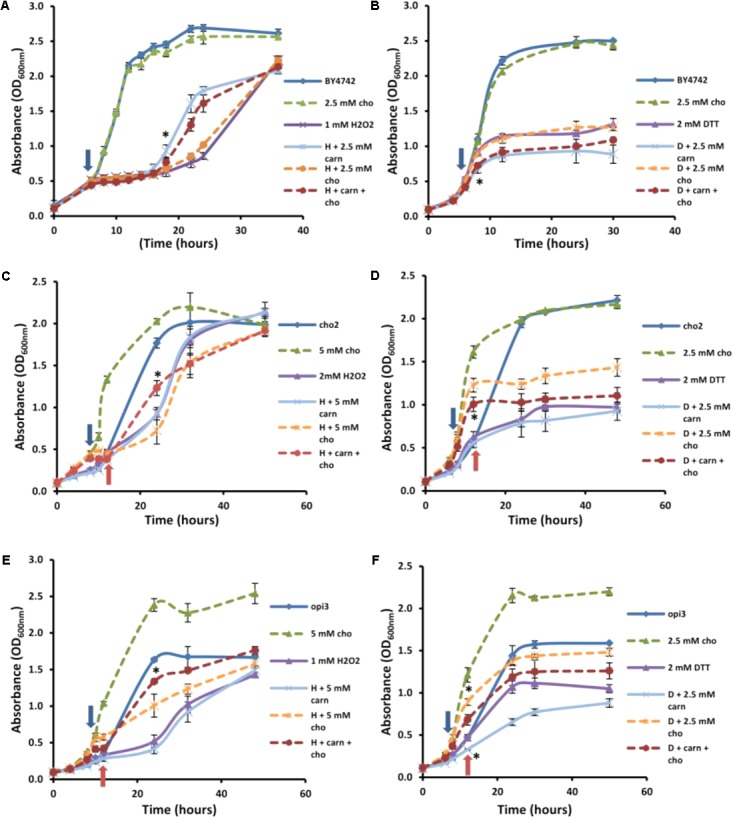
Effect of choline on carnitine phenotypes in the mutant and wild type cells. Broken lines indicate choline-treated cultures. Addition of choline (cho) has no significant effect alone or in combination with carnitine (carn) on either hydrogen peroxide **(A)** or DTT **(B)** in the WT. Choline supplementation restores the effects of carnitine in the mutant Δ*cho2* both in hydrogen peroxide **(C)** and DTT **(D)**. The presence of choline also restores the effect of carnitine in Δ*opi3* treated with hydrogen peroxide **(E)**. However, under these conditions Δ*opi3* was found to be responsive to carnitine together with DTT, irrespective of the presence of choline **(F)**. Arrows represent the timing of addition of DTT or hydrogen peroxide. For the mutants blue arrows indicate the timing of treatment for cultures grown with choline and red arrows for cultures grown without choline. All experiments were done in triplicate with error bars representing standard deviation (*^∗^p* < 0.05 vs. H_2_O_2_ or DTT).

Addition of choline by itself had no effect on the growth of the wild type cultures (**Figure 5**). There was a slight improvement in recovery time for cultures containing choline in the presence of hydrogen peroxide; however, this effect was far less than that observed for carnitine (**Figure 5A**). In addition, when carnitine and choline were added together, in a 1:1 molar ratio, to the same final concentration that the individual compounds were supplemented, the protective effect did not match that of carnitine by itself at the higher concentration. Similarly, choline, supplemented alone or in combination with carnitine did not have any significant effect on the DTT phenotypes (**Figure 5B**).

These conditions were also tested in the mutants. Both mutants responded to hydrogen peroxide and DTT similarly to the wild type, however, Δ*cho2* required a higher concentration of hydrogen peroxide than Δ*opi3* and the wild type (2 mM as opposed to 1 mM) for a significant decrease in growth to be observed (**Figure 5**). The reason for the increased H_2_O_2_ resistance in this strain is uncertain. No protective effect for carnitine was observed for Δ*cho2* and Δ*opi3* when stressed with hydrogen peroxide (**Figures 5C,E**).

As expected, the addition of choline significantly improved the growth of both mutants. Moreover, when choline was present, the protective effect of carnitine in the presence of hydrogen peroxide was restored to WT levels in the case of both phospholipid synthesis mutants. Both mutants also responded similarly to the wild type when stressed with DTT (**Figures 5D,F**). For Δ*cho2*, there was no significant increase in toxicity of DTT when applied in combination with carnitine (**Figure 5D**). This effect of carnitine was, however, restored when choline was also present in the medium. Conversely, in the Δ*opi3* mutant, the effect of DTT was increased in the presence of carnitine regardless of the presence of choline (**Figure 5F**).

### LCMS Analysis of Lipid Composition

#### Comparison of the Phospholipid Composition Between Wild Type and Δ*opi3*, in Different Growth Stages

To provide a comparative analysis of the lipid composition between different strains and treatments, the intensity percentage, calculated as the area of the peak of a given compound divided by the total peak area of all the compounds in the HILIC-ESI-MS chromatogram, was used. The results therefore do not represent changes in the concentration of compounds but in the relative abundance of the compound in the sample. This measure was reported in previous studies to have a reliable and linear relationship to the molar abundance of a compound in a sample (Tarasov et al., 2014).

**FIGURE 6:**
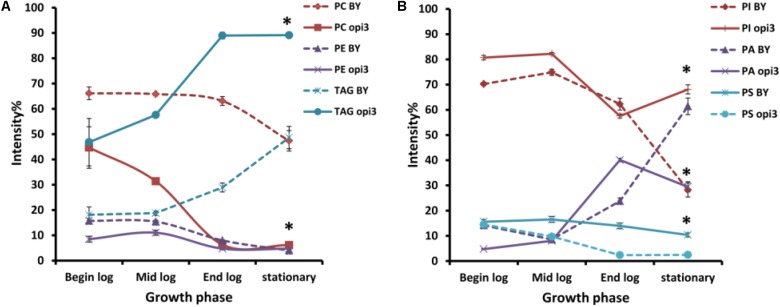
Comparison of the phospholipid and TAG composition of the wild type (BY) and mutant Δ*opi3* cultures during different growth phases observed in positive **(A)** and negative **(B)** ionization mode. All experiments were done in triplicate with error bars representing standard deviation (^∗^*p* < 0.01 vs. BY).

To compare the phospholipid composition of the wild-type with Δ*opi3* at different growth stages and also to examine the effect of choline depletion on the lipidome of cells, both strains were pre-cultured in media containing choline and then transferred to growth medium without choline. The cultures were then sampled at four different growth stages over a period of 30 h (**Figure 6**). As would be expected, the abundance of PC in the mutant cells are less that the wild-type and also decreases faster than observed in the wild-type cultures. The abundance of PE in both strains also decreases as the cultures approached stationary phase. For both the wild type and mutant cultures, the levels of TAG increase toward stationary phase, however, the levels of TAG present in the mutant were always significantly higher than in the wild type reaching up to three fold higher at the end of log phase. PI was also present in significantly higher levels in the mutant than in the wild type at stationary phase with a 40% difference in abundance. An increase in PA was seen for both strains as cultures reached stationary phase and the opposite trend was observed for PS, although this was much more prominent in the mutant than the wild type.

#### Effect of Choline and Carnitine Supplementation on Lipid Composition of Wild Type and Mutant Cells

**FIGURE 7:**
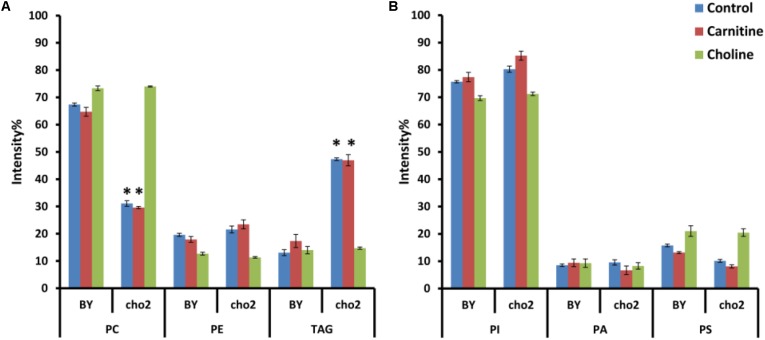
Phospholipid and TAG composition of the mutants alone and in response to carnitine and choline in positive **(A)** and negative **(B)** ionization mode. All experiments were done in triplicate with error bars representing standard deviation (^∗^*p* < 0.001 vs. BY control).

To investigate the effects of choline and carnitine supplementation on the lipidome of wild type and mutant strains, cultures were grown in minimal medium supplemented with choline and then transferred to medium which was without or with either 2 mM choline or 2.5 mM carnitine. The mutant Δ*cho2* showed a reduction in abundance of PC by 36% and also a 34% increase of TAG compared with the wild type (**Figure 7**). Addition of choline led to a reversal of these effects in the mutant, producing a lipid profile similar to that of the wild type. Preliminary results also indicate that Δ*opi3* reacts similarly to Δ*cho2* in response to choline supplementation (data not shown). However, the addition of carnitine did not lead to any significant changes in the phospholipid composition of cells.

The fatty acid composition within the cultures was also analyzed. Although choline supplementation restored the fatty acid profile of Δ*cho2* to that of wild type cultures, carnitine supplementation did not result in any significant changes in the fatty acid profile of the mutant or wild type cultures (data not shown).

#### Effect of Treatment With Hydrogen Peroxide and DTT on Lipid Composition of Cells

It was also investigated whether carnitine treatment results in any changes in the lipid composition of cells after stressing with DTT and hydrogen peroxide. To this end, cultures were grown to an OD_600nm_ of 0.8 in the presence and absence of carnitine, at which point samples were taken for lipid extraction. Cultures were treated with either DTT or hydrogen peroxide and samples were taken for lipid extraction 30 and 60 min after treatment.

Treatment with carnitine by itself did not result in any major shifts in the phospholipid and TAG composition within the cells. Also, no major changes in lipid composition could be seen for cultures treated with hydrogen peroxide or DTT (data not shown).

## Discussion

Previous studies in the model organism, *S. cerevisiae*, have illustrated that L-carnitine impacts cellular survival positively or negatively depending on the type of redox stress. As confirmed by this study, carnitine supplemented to growing yeast cultures has a protective effect against hydrogen peroxide and a detrimental effect when carnitine is combined with DTT (Franken and Bauer, 2010). Although the protective effect of carnitine against oxidative stress has been established by several studies (Franken and Bauer, 2010; Li et al., 2012; Sachan et al., 2012), the detrimental outcome in combination with DTT has thus far only been observed in yeast. The molecular or metabolic foundations behind these phenotypes, however, remain unknown. This study therefore aimed to identify possible genetic elements that are involved in the carnitine phenotypes by executing a screen of the entire collection of viable yeast null-mutants against the two carnitine phenotypes. Three deletion strains were identified in this screen, namely Δ*yap1*, Δ*cho2*, and Δ*opi3*. Of these three mutants, *YAP1* has previously been linked to carnitine-dependent phenotypes (Franken and Bauer, 2010). *OPI3* and *CHO2* encode enzymes that catalyze the final two sequential reactions in the pathway to synthesize phosphatidylcholine from phosphatidylethanolamine, and their co-selection from a screen of a genome-wide deletion strain library which only yielded three mutants strongly suggests a specific connection between these genes and carnitine-related activities.

It is noteworthy that carnitine does show structural chemical similarities to choline, the precursor to the Kennedy pathway, which provides an alternative route to phosphatidylcholine synthesis, and shares the same membrane transporter. However, the data convincingly show that the link between choline and carnitine is not based on some overlapping roles or structural similarities, since choline shows none of the carnitine-specific phenotypic impacts. The data also suggest that the suppression phenotype of the *OPI3* and *CHO2* mutants is not a result of changes in carnitine transport that would be due to changed membrane phospholipid composition. Indeed, deletion of the *HMN1* carnitine transporter-encoding gene did not impact on the carnitine-dependent phenotypes.

The two mutants display a general reduced growth even in the presence of choline when compared to the wild type, suggesting some broader impact on cellular physiology (Mcgraw and Henry, 1989). However, the reduced growth is unlikely to be causatively linked to the suppression of carnitine-dependent phenotypes since many of the mutant strains that were assessed in the screen show similar growth defects, but did not show suppression of carnitine-dependent phenotypes.

The restoration of the protective effect for carnitine against hydrogen peroxide in mutant cultures supplemented with choline, shows that the presence of this compound and/or of phosphatidylcholine is required for this phenotype. This provides the first report of carnitine being dependent on the presence of another metabolite to exert a phenotypic influence.

Liquid Δ*cho2* cultures which were treated with DTT, were unresponsive to carnitine supplementation, except in the presence of choline. However, Δ*opi3* reacted as the WT to the combination of DTT and carnitine in these conditions. This result was contradictory to what was found on plate assays for Δ*opi3*. This difference suggests that the interaction of DTT and carnitine are condition dependent. Furthermore, it is possible that the growth retardation effect of carnitine could, at least partially, be responsible for the increased toxicity observed when carnitine is supplemented in addition to DTT in liquid media.

Previous studies have suggested that the composition of the cell membrane influences the susceptibility of the cell to adverse environmental conditions (Rodríguez-Porrata et al., 2011; De Smet et al., 2013; Henderson et al., 2013). Changes in cellular lipid composition in the mutants and in the wild type in response to the different treatments were therefore investigated to establish if the link found between the carnitine shuttle-independent phenotypes and Δ*cho2* and Δ*opi3* are indicative of a deeper requirement for membrane lipid homeostasis. A decrease in PC abundance in the mutant cultures was observed and became more prominent as biomass increased. The data show that the progressive decline in the availability of this major phospholipid resulted in increased PI and TAG abundance. These changes in phospholipid composition had been reported previously and are thought to aid in the maintenance of proper membrane structure and function in the absence of PC (Boumann et al., 2006; Malanovic et al., 2008).

Treatment with DTT and hydrogen peroxide did not to lead to changes in the lipid profile of cultures. This is in contrast to results from a study in red blood cells which indicated that oxidative stress resulted in decreases in PS and increases in PC (Freikman et al., 2008). However, this response was thought to be a strategy to avoid phagocytosis and would therefore not be expected to be of physiological relevance in yeast. In addition, carnitine did not induce changes in the lipid profile in the wild type or mutant cells and no effect for carnitine supplementation on the lipid composition of cells stressed with hydrogen peroxide or DTT was found. This indicates that alteration of lipid composition is likely not linked to the phenotypic effects of carnitine supplementation.

It is clear from these results that the deletion of the genes encoding for the methyltransferases which are involved in the synthesis of PC from PE result in wide spread changes within the lipidome. However, deletion of *CHO2* and *OPI3* could also lead to other changes within the cell which have not been considered in this work (Summers et al., 1988). The specific changes or combination of changes which lead to the decreased responsiveness of these strains to carnitine remains unknown. However, the fact that deletion of these two enzymes, to the exclusion of others which may lead to similar effects, for example, other mutants displaying the opi^-^ phenotype, but do not display decreased responsiveness to carnitine, strongly argue that loss of phosphatidylcholine is in some way involved in these effects. The loss of PC, however, results in a large range of different alterations in cellular lipid composition, any combination of which could be involved in this response. Further investigation is certainly required to determine the mechanisms behind the action of carnitine and the cellular processes involved in this response.

## Author Contributions

MdP carried out most of the research, contributed to experimental planning and data analysis, and co-wrote the first draft of the manuscript. JF carried out part of the mutant screen, contributed to experimental planning, data analysis and interpretation and co-wrote and edited the manuscript. FB conceptualized the project, contributed to experimental planning, data analysis and data interpretation, and edited the final manuscript.

## Conflict of Interest Statement

The authors declare that the research was conducted in the absence of any commercial or financial relationships that could be construed as a potential conflict of interest.

## Footnotes

1 http://www.euroscarf.de/index.php?name=Contact
